# M. Raciborski on the Physiology of Menstruation

**Published:** 1842-03

**Authors:** 


*M. Raciborski on the Physiology of Menstruation.*—Our readers will remember that in our recent review of Gendrin's *Traite Philosophique*, we drew their attention to this curious and hitherto ill-understood subject of physiological inquiry. From the researches of this gentleman, and from those of M. Negrier, as well as of Dr. Robert Lee, it was suggested that there is an actual rupture of one of the ovarian vesicles at each period of menstruation, and that the sanguineous discharge from the uterus was the result of this lesion. M. Raciborski questions the accuracy of this statement. While he admits that the primary movement in each act of menstruation is a congestion of the vessels of the ovaries, he denies that any rupture of their surface necessarily takes place at the same time.

M. Raciborski sums up the conclusions to which he has come, after a very elaborate inquiry, in the following propositions:—

1. That menstruation is a consequence of the accomplishment of the development of the ovaries.

2. That it is the direct result of the means employed by nature to place the ends of the Fallopian tubes and the ovaries in the relations necessary to fecundation and the passage of the fecundated ova.

3. That the sanguineous congestion, which is indispensable for obtaining those conditions in the human being, appears sufficient in itself to explain the occurrence of the hæmorrhage which constitutes menstruation—without having recourse to supposing that there is any necessary solution of continuity.

4. That the vertical position, favoring still more the effects of sanguineous congestion on the generative organs, may be one of the principal reasons of the abundance of the menstrual flux in women, and in some species of simiæ.

5. That, for want of having precise information as to the nature and theory of menstruation, it has been hitherto impossible to establish a rational treatment of the various disorders induced by irregularities of this function.

6. That it is not yet sufficiently proved that the ovula arrive successively to maturity at each menstrual epoch, or that the most mature ovum then approaches nearest the surface of the ovarium, there to become ruptured and give exit to a germ.—*Med. Chir. Rev. Oct.* 1841, *from L'Experience.*

